# Supplementary material for: Quality of life in the postpartum period of Mexican women living with HIV: The role of clinical and sociodemographic factors
Source: PLoS One. 2026 May 14;21(5):e0330790. doi: 10.1371/journal.pone.0330790 (PMC13175498; doi:10.1371/journal.pone.0330790)
Supplement: S1 File — (DOCX) [file pone.0330790.s001.docx]

**SUPPLEMENTARY MATERIAL 1.** WHOQoL-HIV Bref Instrument (Adapted version)

**ASSESSMENT OF QUALITY OF LIFE (QoL) IN THE POSTNATAL PERIOD AMONG MEXICAN WOMEN LIVING WITH HIV WHO ARE RECEIVING ANTIRETROVIRAL THERAPY**

Thank you for agreeing to participate in this survey, which is designed to assess your perception of your quality of life during the postnatal period.

I am one of the data collectors for this study and will be with you to ask you a series of questions. This questionnaire should take about **30 minutes** to complete, and all the answers you provide will be treated **confidentially**.

This questionnaire is part of a clinical research protocol approved by the Research, Ethics, and Biosafety Committees of the **National Institute of Perinatology**, registration number **212250-3120771**. This study will allow us to assess your quality of life (regardless of HIV infection) and its relationship to the proper use of your antiretroviral medications.

**The primary goal** is to reduce the barriers and factors contributing to non-adherence to your medication regimen by establishing viable medical interventions. The analyzed and interpreted results will be communicated to the relevant staff at the Institute so that the necessary interventions can be carried out.

**PERSONAL INFORMATION**

Before we begin, we would like to ask you some general questions about yourself. Please mark the most appropriate answer or fill in the corresponding space.

**SOCIODEMOGRAPHIC DATA**

**Name:** __________________________________

**File number No.:**_________________

**How old are you?** ________________ (age in years)

**What is your highest level of education completed?** __________ *(None / Elementary-Middle School / High School / Higher Education)*

**What is your marital status?** *(Single / Married / Common-law union / Separated / Divorced / Widow)*

**Occupation:** __________________ *(Permanent / Contract / Unemployed)*

**Average monthly income?** ____________________ Pesos

**Place of residence?** *(Urban area / Rural area)*

**Do you have any chronic illnesses?** Yes / No

**If you answered "Yes," which of the following chronic illnesses do you have?** *(Hypertension (HTN) / Diabetes Mellitus (DM) / Epilepsy / Tuberculosis (TB) / Other, please specify: __________*

**How do you consider your state of health?** *(Very poor / Poor / Neither good nor poor / Good / Very good)*

**Do you currently feel ill?** Yes / No

**If you have or feel any discomfort, what do you think it is?** _______________________

**Please answer the following questions if they apply to you:**

**What is your HIV status?** *(Asymptomatic / Symptomatic / Advanced)*

**What is the estimated date you believe you were infected?**  ______________________

**In what year did you have your first positive HIV test?** ______________________

**What do you think was the route of infection?** (Circle only one option) *(Sexual intercourse / Injection drug use / Blood transfusion / Vertical (mother-to-child) / Other (specify): _________________)*

**Immunological values:**

- **Initial CD4 count:** _________ **Current CD4 count:** ____________
- **Initial Viral Load:** _________ **Current Viral Load:** ____________

**What ARV (antiretroviral) treatment are you receiving?** ____________________________

**How many tablets do you have left now?** ____________________

**How many tablets do you take per day?** ________________________

**In addition to ARV treatment, do you take any other medication or vitamins?** Yes / No

Which medication? ____________________ How many tablets per day? _________________

**How many months pregnant are you?** ______________ **What was the date of your last menstrual period?** ______________

**If you have already given birth, how many months old is your child?** ___________

**Did you have any complications during pregnancy or at the time of delivery/C-section?** _______________________________________________________________

**Have you been diagnosed with or tested positive for COVID-19?** ___________________________

**Do you have the support of your family and/or partner?** ________

What does this support consist of? ___________________________________________________

**INSTRUCTIONS:**

The following statements are intended to find out how you feel about your quality of life, your health, and other areas of your life. Please answer all questions. If you are unsure which answer to give, please choose the one that seems most appropriate. This is often your first response. Consider your standards, hopes, pleasures, and concerns. We intend to ask you what you think about your life **in the last two weeks**. For example, thinking about the last two weeks, a question might be:

|  | Not at all | A little | A moderate  amount | Very much | Extremely |
| --- | --- | --- | --- | --- | --- |
| How well are you able to concentrate? | 1 | 2 | 3 | 4 | 5 |

You should circle the number that best fits how well are you able to concentrate over the last two weeks. So you would circle the number 4 if you were able to concentrate very much. You would circle number 1 if you were not able to concentrate at all in the last two weeks.

**Please read each question, assess your feelings, and circle the number on the scale for each question that gives the best answer for you.**

|  | Very poor | Poor | Neither poor  nor good | Good | Very good |
| --- | --- | --- | --- | --- | --- |
| How wo uld you rate your quality of life? | 1 | 2 | 3 | 4 | 5 |

|  | Very  dissatisfied | Dissatisfied | Neither  satisfied nor  dissatisfied | Satisfied | Very  satisfied |
| --- | --- | --- | --- | --- | --- |
| How satisfied are you with your health? | 1 | 2 | 3 | 4 | 5 |

The following questions ask about **how much** you have experienced certain things in the last two weeks.

|  | Not at all | A little | A moderate  amount | Very much | Extremely |
| --- | --- | --- | --- | --- | --- |
| To what extent do you feel that physical pain  prevents you from doing what you need to  do? | 1 | 2 | 3 | 4 | 5 |
| How much are you bothered by any physical  problems related to your HIV infection? | 1 | 2 | 3 | 4 | 5 |
| How much do you need any medical treatment to function in your daily life? | 1 | 2 | 3 | 4 | 5 |
| How much do you enjoy life? | 1 | 2 | 3 | 4 | 5 |
| To what extent do you feel your life to be  meaningful? | 1 | 2 | 3 | 4 | 5 |
| To what extent are you bothered by people  blaming you for your HIV status | 1 | 2 | 3 | 4 | 5 |
| How much do you fear the future? | 1 | 2 | 3 | 4 | 5 |
| How much do you worry about death? | 1 | 2 | 3 | 4 | 5 |

|  | Not at all | A little | A moderate  amount | Very much | Extremely |
| --- | --- | --- | --- | --- | --- |
| How well are you able to concentrate? | 1 | 2 | 3 | 4 | 5 |
| How safe do you feel in your daily life? | 1 | 2 | 3 | 4 | 5 |
| How healthy is your physical environment? | 1 | 2 | 3 | 4 | 5 |

The following questions ask about **how completely** you experience or were able to do certain things in the last two weeks.

|  | Not at all | A little | A moderate  amount | Very much | Extremely |
| --- | --- | --- | --- | --- | --- |
| Do you have enough energy for every day  life? | 1 | 2 | 3 | 4 | 5 |
| Are you able to accept your bodily appearance? | 1 | 2 | 3 | 4 | 5 |
| Have you enough money to meet your needs? | 1 | 2 | 3 | 4 | 5 |
| To what extent do you feel accepted by the people you know? | 1 | 2 | 3 | 4 | 5 |
| How available to you is the information that you need in your day-to-day life? | 1 | 2 | 3 | 4 | 5 |
| To what extent do you have the opportunity for leisure activities? | 1 | 2 | 3 | 4 | 5 |

|  | Very poor | Poor | Neither poor, nor good | Good | Very good |
| --- | --- | --- | --- | --- | --- |
| How well are you able to get around? | 1 | 2 | 3 | 4 | 5 |

The following questions ask you how **good or satisfied** you have felt about various aspects of your life over the last two weeks.

|  | Very  dissatisfied | Dissatisfied | Neither satisfied nor dissatisfied | Satisfied | Very satisfied |
| --- | --- | --- | --- | --- | --- |
| How satisfied are you with your sleep? | 1 | 2 | 3 | 4 | 5 |
| How satisfied are you with your ability to perform your daily living activities? | 1 | 2 | 3 | 4 | 5 |
| How satisfied are you with your capacity for work? | 1 | 2 | 3 | 4 | 5 |
| How satisfied are you with yourself? | 1 | 2 | 3 | 4 | 5 |
| How satisfied are you with your personal relationships? | 1 | 2 | 3 | 4 | 5 |
| How satisfied are you with your sex life? | 1 | 2 | 3 | 4 | 5 |
| How satisfied are you with the support you get from your friends? | 1 | 2 | 3 | 4 | 5 |
| How satisfied are you with the conditions of your living place? | 1 | 2 | 3 | 4 | 5 |
| How satisfied are you with your access to health services? | 1 | 2 | 3 | 4 | 5 |
| How satisfied are you with your transport? | 1 | 2 | 3 | 4 | 5 |

The following question refers to **how often** you have felt or experienced certain things in the last two weeks.

|  | Never | Seldom | Quite often | Very often | Always |
| --- | --- | --- | --- | --- | --- |
| How often do you have negative feelings such as blue mood, despair, anxiety, depression? | 1 | 2 | 3 | 4 | 5 |

Did someone help you to fill out this form?

_______________________________

How long did it take to fill this form out? _________________________________

Do you have any comments about the assessment? ______________________________________________________________________________________________________________________________

_______________________________________________________________

**THANK YOU FOR YOUR HELP**
