# Supplementary material for: Quality of life in the postpartum period of Mexican women living with HIV: The role of clinical and sociodemographic factors
Source: PLoS One. 2026 May 14;21(5):e0330790. doi: 10.1371/journal.pone.0330790 (PMC13175498; doi:10.1371/journal.pone.0330790)
Supplement: S1 Table — (DOCX) [file pone.0330790.s002.docx]

**S1 Table.** Comparison of the total QoL median score and their domains in the Mexican postpartum WLWH using three different combinations of ART

| ***Domains*** | **Antiretroviral Therapy** | | | |
| --- | --- | --- | --- | --- |
|  | ***Group A***  ***Median [IQR]***  ***n= 17*** | ***Group B***  ***Median [IQR]***  ***n= 8*** | ***Group C***  ***Median [IQR]***  ***n= 45*** | ***P value*** |
| **I. Physical health** | 17 [16-18] | 16 [14-16] | 15 [12-17] | **0.008*** |
| **II. Psychological health** | 15 [14-17.5] | 12 [11-18] | 14 [13-16] | 0.169 |
| **III. Independence level** | 17 [15-18] | 17 [15-18] | 15 [13-17.5] | 0.159 |
| **IV. Social relationships** | 15 [11-16] | 14 [13-16] | 16 [14-17] | 0.078 |
| **V. Environment** | 15 [13-17] | 15 [14-16] | 14 [13-16] | 0.659 |
| **VI. Spirituality, religion and personal beliefs** | 14 [12.5-16.5] | 11 [8-16] | 13 [10-16] | 0.241 |
| **Total QoL** | 15 [14.5-16.5] | 14 [13-16] | 14 [13-16] | **0.039*** |

**Antiretroviral therapy.** Group A: 2 nucleoside reverse transcriptase inhibitors (NRTIs) + 1 non-nucleoside reverse transcriptase inhibitor (NNRTI). Group B: 2 NRTIs + 1 protease inhibitors (PI). Group C: 2 NRTIs + 1 integrase inhibitors (II).

Median [Interquartile range], Kruskal‒Wallis test, *p-*value <0.05.
