# Supplementary material for: Quality of life in the postpartum period of Mexican women living with HIV: The role of clinical and sociodemographic factors
Source: PLoS One. 2026 May 14;21(5):e0330790. doi: 10.1371/journal.pone.0330790 (PMC13175498; doi:10.1371/journal.pone.0330790)
Supplement: S2 Table — (DOCX) [file pone.0330790.s003.docx]

**S2 Table.** Comparison of the total QoL median score and their domains at different postpartum times and in different years of interviews of the Mexican postpartum WLWH

| ***Domains*** | ***Pospartum times*** | | | ***Years of interviews*** | | | |
| --- | --- | --- | --- | --- | --- | --- | --- |
|  | ***Less 1 year***  ***n=55*** | ***Over 1 year***  ***n=20*** | ***P-value^a^*** | ***2020***  ***n= 20*** | ***2021***  ***n= 48*** | ***2022***  ***n= 7*** | ***P-value^b^*** |
| **I. Physical health** | 16 [12-17] | 17 [15-18] | ***0.035**** | 16 [14.25-18] | 16 [14-18] | 10 [8-11] | ***<0.001*** |
| **II. Psychological health** | 14 [14-16] | 14 [11.25-17.75] | 0.566 | 14 [12.25-15] | 14 [13-17] | 14 [14-16] | 0.619 |
| **III. Independence level** | 16 [14-17] | 16.5 [14.25-18] | 0.118 | 16 [14-17] | 16 [15-18] | 13 [12-18] | 0.130 |
| **IV. Social relationships** | 16 [13-17] | 13 [12-16] | ***0.011**** | 12 [11-13] | 16 [15-16] | 18 [18-20] | ***>0.001*** |
| **V. Environment** | 14 [13-16] | 15 [12.25-16.75] | 0.851 | 15.5 [13-17] | 14 [13-16] | 15 [14-16] | 0.324 |
| **VI. Spirituality, religion and personal beliefs** | 14 [10-16] | 13 [10.25-15.75] | 0.609 | 11.5 [10-14] | 14 [12-17] | 10 [9-12] | ***0.017*** |
| **Total QoL** | 15 [13-16] | 14.5 [13-17] | 0.493 | 14.5 [13-16] | 15 [13-16] | 13 [13-14] | 0.131 |

WLWH: Women living with HIV.

^a^ Median [Interquartile range]. U Mann-Whitney’s test, *p*-value <0.05.

^b^ Median [Interquartile range]. Kruskal-Wallis test, *p*-value <0.05.
