# Supplementary material for: Quality of life in the postpartum period of Mexican women living with HIV: The role of clinical and sociodemographic factors
Source: PLoS One. 2026 May 14;21(5):e0330790. doi: 10.1371/journal.pone.0330790 (PMC13175498; doi:10.1371/journal.pone.0330790)
Supplement: S3 Table — (DOCX) [file pone.0330790.s004.docx]

**S3 Table.** Spearman's correlation coefficients between demographic and non-pathological history, infectious history, ART management, obstetric history and sexual behavior *vs* total QoL score and their domains in the Mexican postpartum WLWH

| **Variables** | **Physical health** | **Psychological health** | **Independence level** | **Social relationships** | **Environment** | **Spirituality, religion and personal beliefs** | **Total**  **QoL** |
| --- | --- | --- | --- | --- | --- | --- | --- |
| **Year of interview** | **-.286^*^** | .107 | -.101 | **.826^**^** | -.074 | .065 | -.060 |
| **Age** | .122 | .177 | .182 | .053 | -.011 | .136 | .175 |
| **Educational status** | -.108 | -.127 | **-.233^*^** | .069 | .074 | -.053 | -.087 |
| **Marital status** | .175 | .093 | .126 | **-.237^*^** | -.040 | -.159 | .004 |
| **Employment** | .040 | -.200 | -.106 | -.108 | -.099 | -.193 | -.179 |
| **Employment type** | .068 | .159 | .049 | -.105 | -.189 | .187 | .022 |
| **Monthly income** | .050 | **.243^*^** | .074 | .076 | .121 | .079 | .150 |
| **Place of residence** | -.054 | .141 | .122 | .033 | .101 | .008 | .091 |
| **Other chronic diseases** | -.075 | -.135 | .019 | .133 | -.169 | -.154 | -.136 |
| **Type of chronic diseases** | 0.036 | .096 | .025 | -.185 | .061 | .081 | .041 |
| **HIV symptoms** | -.087 | **-.230^*^** | **-.229^*^** | .014 | -.143 | -.094 | -.203 |
| **Years since the HIV diagnosis** | .095 | .178 | .166 | -.119 | .090 | -.046 | .125 |
| **Initial CD4 count at HIV diagnosis** | .154 | .172 | .181 | -.151 | .050 | -.029 | .154 |
| **Last CD4 count during pregnancy** | .174 | **.262^*^** | .192 | .140 | .128 | .150 | .218 |
| **Initial viral Load valor** | -.104 | -.159 | -.174 | -.189 | -.097 | -.147 | -.177 |
| **Initial viral Load determination** | -.089 | -.070 | -.058 | .103 | -.043 | .040 | -.086 |
| **Current viral load valor** | .050 | .184 | .256 | -.430 | -.104 | -.120 | .123 |
| **Current viral load determination** | -.093 | -.090 | -.120 | .189 | .014 | .189 | .011 |

Interpretative results of the total QoL and their domains: Spearman Rho (ρ), *p*-value * <0.05 and ** <0.001

**S3 Table.** Spearman's correlation coefficients between demographic and non-pathological history, infectious history, ART management, obstetric history and sexual behavior *vs* total QoL score and their domains in Mexican postpartum WLWH (*continued*...)

| **Variables** | **Physical health** | **Psychological health** | **Independence level** | **Social relationships** | **Environment** | **Spirituality, religion and personal beliefs** | **Total**  **QoL** |
| --- | --- | --- | --- | --- | --- | --- | --- |
| **ART therapy during prenatal care** | **-.313^**^** | -.050 | -.197 | **.260^*^** | -.082 | -.042 | -.212 |
| **ART therapy by category during prenatal care** | **-.330^**^** | -.153 | -.223 | .195 | -.096 | -.135 | **-.272^*^** |
| **ART therapy during postpartum** | **-.425^**^** | -.086 | -.233 | **.294^*^** | -.082 | .015 | -.234 |
| **ART therapy by category during postpartum** | **-.378^**^** | -.148 | -.230 | **.261^*^** | -.111 | -.073 | **-.289^*^** |
| **Time of postpartum** | .196 | .004 | .217 | **-.440^**^** | .001 | -.060 | .057 |
| **Gestational complications** | .111 | .022 | .106 | -.168 | .223 | .051 | .134 |
| **Current birth delivery** | -.100 | .059 | .005 | .159 | -.157 | -.039 | -.117 |
| **COVID-positive diagnosis** | .228 | .096 | -.025 | -.044 | -.031 | .080 | .073 |
| **Partner or family support** | -.006 | .100 | -.019 | .164 | .193 | .000 | .118 |
| **Type of partner or family support** | .027 | .128 | .090 | .187 | -.009 | -.021 | .065 |
| **Gestational age at medical admission** | .101 | -.013 | .081 | **-.591^**^** | -.037 | .064 | .028 |
| **Age of the beginning of sexual life** | .072 | **.259^*^** | .126 | -.051 | -.009 | .213 | .142 |
| **Number of sexual partners** | -.171 | -.227 | **-.417^**^** | .084 | -.023 | .016 | -.180 |
| **Sexual abuse history** | .218 | .048 | .062 | .106 | **.231^*^** | .064 | .176 |
| **Perceived HIV transmission route** | .132 | .120 | .168 | -.103 | .144 | -.086 | .109 |
| **Current partner HIV-status** | -.137 | **-.253^*^** | -.169 | **-.260^*^** | -.080 | -.078 | -.204 |

Interpretative results of the total QoL and their domains: Spearman Rho (ρ), *p*-value * <0.05 and ** <0.001

**S3 Table.** Spearman's correlation coefficients between demographic and non-pathological history, infectious history, ART management, obstetric history and sexual behavior *vs* total QoL score and their domains in the Mexican postpartum WLWH (*continued*...)

| **Variables** | **Physical health** | **Psychological health** | **Independence level** | **Social relationships** | **Environment** | **Spirituality, religion and personal beliefs** | **Total**  **QoL** |
| --- | --- | --- | --- | --- | --- | --- | --- |
| **Gestations** | .065 | .212 | **.282^*^** | .119 | -.182 | .225 | .146 |
| **Abortions** | -.051 | -.044 | .029 | .154 | -.131 | .106 | -.008 |
| **Vaginal delivery** | .209 | -.044 | .087 | .220 | -.023 | .071 | .054 |
| **Caesarean sections** | -.077 | .015 | .078 | **-.230^*^** | .007 | -.012 | .005 |
| **Previous HIV-infected children** | .001 | -.169 | -.174 | -.122 | .034 | -.115 | -.123 |
| **Smoking habit** | -.116 | -.129 | -.163 | **-.325^**^** | .032 | -.136 | -.172 |
| **Alcoholism and drug addiction** | -.125 | -.091 | -.150 | .163 | -.013 | -.137 | -.118 |
| **Partner's addictions** | .195 | -.167 | .095 | -.200 | .121 | -.106 | .086 |
| **Tattoos and piercings** | -.239 | **-.280^*^** | -.169 | .139 | .074 | **-.321^*^** | -.219 |
| **Efavirenz during pregnancy** | **-.275^*^** | -.182 | -.178 | .156 | -.069 | -.155 | **-.263^*^** |
| **Place where they come from** | -.0530 | .0270 | -.022 | .106 | -.220 | .046 | -.106 |
| **Adherence to ART therapy** | -.016 | .076 | .051 | -.134 | -.035 | .135 | .087 |
| **Intrapartum ART prophylaxis** | -.058 | .125 | .050 | .212 | -.077 | -.068 | -.060 |

Interpretative results of the total QoL and their domains: Spearman Rho (ρ), *p*-value * <0.05 and ** <0.001
