# Supplementary material for: Quality of life in the postpartum period of Mexican women living with HIV: The role of clinical and sociodemographic factors
Source: PLoS One. 2026 May 14;21(5):e0330790. doi: 10.1371/journal.pone.0330790 (PMC13175498; doi:10.1371/journal.pone.0330790)
Supplement: S4 Table — (DOCX) [file pone.0330790.s005.docx]

**S4 Table.** Results of Principal Component Analysis (PCA): Variables and their categories that account for the most total variance in classification into low, medium or high QoL

| **Low QoL** | | | |  | **Medium QoL** | | | |  | **High QoL** | | | |
| --- | --- | --- | --- | --- | --- | --- | --- | --- | --- | --- | --- | --- | --- |
| **Component** | **Variables accounted for most of the variance and category (n=37)** | **Global frequency (n=75)** | **Absolut loading values** |  | **Component** | **Variables accounted for most of the variance and category (n=15)** | **Global frequency (n=75)** | **Absolut loading values** |  | **Component** | **Variables accounted for most of the variance and category (n=23)** | **Global frequency (n=75)** | **Absolut loading values** |
| 1 | Time of postpartum: 3 months | 16.2% | -0.756 |  | 1 | ART therapy by category during prenatal care: two NRTIs + II | 57.1% | 0.915 |  | 1 | ART therapy by category during prenatal care= two NRTIs + II | 56.5% | 0.849 |
|  | Year of interview: 2021 | 56.8% | 0.734 |  |  | ART therapy by category during postpartum= two NRTIs + II | 53.8% | 0.9 |  |  | ART therapy by category during postpartum= two NRTIs + II | 54.5% | 0.801 |
|  | ART therapy by category during postpartum: two NRTIs + II | 76.5% | 0.713 |  |  | Year of interview= 2021 | 66.7% | 0.896 |  |  | Efavirenz during pregnancy= No | 63.6% | 0.77 |
|  | VI. Spirituality, religion and personal beliefs (QoL score): 15 | 18.9% | 0.69 |  |  | Efavirenz during pregnancy= No | 66.7% | 0.88 |  |  | ART therapy during prenatal care= RAL + FTC + TDF | 39.1% | 0.735 |
|  | I. Physical health (QoL score)= 14 | 16.2% | -0.683 |  |  | ART therapy during prenatal care= FTC + TDF + EFV or RAL + FTC + TDF | 35.7% each | 0.823 |  |  | Current viral Load_det= Indetectable | 76.2% | 0.704 |
|  | ART therapy by category during prenatal care= two NRTIs + II | 78.4% | 0.631 |  |  | Gestations= 2 and 3 | 26.7% each | 0.767 |  |  | ART therapy during postpartum= RAL + FTC + TDF | 40.9% | 0.627 |

**S4 Table.** Results of Principal Component Analysis (PCA): Variables and their categories that account for the most total variance in classification into low, medium or high QoL (*Continued…*)

| **Low QoL** | | | |  | **Medium QoL** | | | |  | **High QoL** | | | |
| --- | --- | --- | --- | --- | --- | --- | --- | --- | --- | --- | --- | --- | --- |
| **Component** | **Variables accounted for most of the variance and category (n=37)** | **Global frequency (n=75)** | **Absolut loading values** |  | **Component** | **Variables accounted for most of the variance and category (n=15)** | **Global frequency (n=75)** | **Absolut loading values** |  | **Component** | **Variables accounted for most of the variance and category (n=23)** | **Global frequency (n=75)** | **Absolut loading values** |
| 2 | QoL self-perception by WLWH (QoL score) = 14 | 29.7% | 0.629 |  | 1 | ART therapy during postpartum= RAL + FTC + TDF | 46.2% | 0.696 |  | 1 | Initial viral Load_det= Indetectable | 55.0 % | 0.608 |
| 4 | Previous HIV-infected children= No | 70.3% | -0.726 |  |  | IV. Social relationships (QoL score)= 16 | 26.7% | 0.688 |  | 2 | VI. Spirituality, religion and personal beliefs (QoL score)= 14 | 17.4% | 0.75 |
|  | Age (years)= 28-31 | 40.5% | 0.672 |  |  | Place where they come from= Mexico City | 53.3% | 0.649 |  |  | Tattoos and piercings: None | 72.2% | -0.736 |
|  | Gestations= 2 | 37.8% | 0.616 |  |  | V. Environment (QoL score)= 14 | 33.3% | -0.631 |  |  | Smoking habit= No | 60.9% | -0.723 |
| 5 | Type of partner or family support = Partner (finalcial/ emotional) | 27.8% | 0.658 |  | 2 | Last CD4 count during pregnancy (cells/mm^3^)= value | value | -0.664 |  |  | IV. Social relationships (QoL score)= 16 | 43.5% | 0.712 |
|  | IV. Social relationships (QoL score)= 14 | 21.6% | -0.623 |  |  | Employment= Housewife | 80% | 0.651 |  |  | Year of interview= 2021 | 73.9% | 0.652 |
| 7 | Sexual abuse history= No | 88.9% | 0.616 |  |  | Current birth delivery= Cesarean section | 80% | 0.648 |  | 3 | Gestations= 3 | 34.8% | 0.725 |
|  |  |  |  |  |  | Smoking habit=No | 73.3% | 0.607 |  |  | Partner's addictions= None | 47.1% | 0.615 |

**S4 Table.** Results of Principal Component Analysis (PCA): Variables and their categories that account for the most total variance in classification into low, medium or high QoL (*Continued…*)

| **Low QoL** | | | | |  | **Medium QoL** | | | |  | **High QoL** | | | |
| --- | --- | --- | --- | --- | --- | --- | --- | --- | --- | --- | --- | --- | --- | --- |
| **Component** | **Variables accounted for most of the variance and category (n=37)** | **Global frequency (n=75)** | **Absolut loading values** | |  | **Component** | **Variables accounted for most of the variance and category (n=15)** | **Global frequency (n=75)** | **Absolut loading values** |  | **Component** | **Variables accounted for most of the variance and category (n=23)** | **Global frequency (n=75)** | **Absolut loading values** |
|  |  |  | |  |  | 3 | Type of partner or family support= Partner (finalcial/ emotional) | 46.7% | 0.758 |  | 3 | Age of the beginning of sexual life (years)= 18 | 23.8% | -0.612 |
|  |  |  | |  |  |  | Gestational age at medical admission= 38,4 weeks | 20% | -0.687 |  |  | Gestational complications= No | 73.9% | -0.607 |
|  |  |  | |  |  |  | Tattoos and piercings=None | 72.7% | 0.673 |  |  | Current viral Load_value (cells/mm^3^)= value | value | 0.606 |
|  |  |  | |  |  |  | Marital status= Cohabiting | 60% | 0.649 |  | 6 | Sexual abuse history= No | 95.7% | -0.617 |
|  |  |  | |  |  | 4 | V. Environment (QoL score)= 14 | 33.3% | -0.607 |  |  |  |  |  |
|  |  |  | |  |  |  | I. Physical health (QoL score)= 16 | 40% | 0.767 |  |  |  |  |  |
|  |  |  | |  |  | 5 | Age (years)= 29 | 20.0% | 0.671 |  |  |  |  |  |
|  |  |  | |  |  |  | QoL self-perception by WLWH (QoL score)= 16 | 73.3% | 0.636 |  |  |  |  |  |

**S4 Table.** Results of Principal Component Analysis (PCA): Variables and their categories that account for the most total variance in classification into low, medium or high QoL (*Continued…*)

| **Low QoL** | | | |  | **Medium QoL** | | | |  | **High QoL** | | | |
| --- | --- | --- | --- | --- | --- | --- | --- | --- | --- | --- | --- | --- | --- |
| **Component** | **Variables accounted for most of the variance and category (n=37)** | **Global frequency (n=75)** | **Absolut loading values** |  | **Component** | **Variables accounted for most of the variance and category (n=15)** | **Global frequency (n=75)** | **Absolut loading values** |  | **Component** | **Variables accounted for most of the variance and category (n=23)** | **Global frequency (n=75)** | **Absolut loading values** |
|  |  |  |  |  | 6 | Intrapartum ART prophylaxis= Yes | 66.7% | 0.763 |  |  |  |  |  |
|  |  |  |  |  | 7 | Alcoholism and drug addiction= None | 60.0% | -0.848 |  |  |  |  |  |
|  |  |  |  |  |  | Partner or family support= Partner | 53.3% | -0.649 |  |  |  |  |  |
|  |  |  |  |  | 8 | Number of sexual partners= 2 | 26.7% | -0.697 |  |  |  |  |  |
