# Supplementary material for: Quality of life in the postpartum period of Mexican women living with HIV: The role of clinical and sociodemographic factors
Source: PLoS One. 2026 May 14;21(5):e0330790. doi: 10.1371/journal.pone.0330790 (PMC13175498; doi:10.1371/journal.pone.0330790)
Supplement: Attachment — Submitted filename: Authors Final Response To Reviewer Comments.pdf [file pone.0330790.s006.pdf]

March 17th, 2026.

Marwan Salih Al-Nimer, MD, PhD
Academic Editor
PLOS One

Dear Editor and Reviewers,

We would like to express our sincere gratitude to the reviewers for their valuable comments on the manuscript entitled **"Quality of life in the postpartum period of mexican women living with HIV: The role of clinical and sociodemographic factors"** by **Mauricio Domínguez-Castro and Alicia Ramírez-Ramírez, *et al*.**, as the first authors. Your comments were all very helpful in improving the manuscript, and we have incorporated your suggestions into our revision. We have responded to each comment raised by the academic editor and reviewers and addressed them accordingly.

According to journal requirements, we request that you to modify the financial disclosure at point 2. Also, we request modifications in the authors' list to add PhD. Edna Basilio-Gálvez; her contribution in the advanced statistical analysis was fundamental for reaching the objectives requested by reviewers.

We hope our manuscript is now suitable for publication in the **PLOS One.**

We look forward to hearing from you at your earliest convenience.

Sincerely yours

MSc. Jessica Hernández Pineda
Department of Infectology and Immunology
National Institute of Perinatology
Phone:+015555209900, ext.497
jesspinq@yahoo.com.mx

---- Journal Requirements ----

When submitting your revision, we need you to address these additional requirements.

1. Please ensure that your manuscript meets PLOS ONE's style requirements, including those for file naming.

Answer. According to your comments, we have reviewed and ensured that this revised version of our manuscript meets PLOS ONE´s requirements.

2. Thank you for stating the following financial disclosure:

“This study was supported by the Instituto Nacional de Perinatología (Mexico City) under approval number 212250-3120771.”

Please state what role the funders took in the study. If the funders had no role, please state: "The funders had no role in study design, data collection and analysis, decision to publish, or preparation of the manuscript." Please include this amended Role of Funder statement in your cover letter; we will change the online submission form on your behalf.

Answer. We wish to include the following in the financial disclosure as follows:

This study was supported by the Instituto Nacional de Perinatología (Mexico City) under approval number 212250-3120771. The founders had no role in study design, data collection and analysis, decision to publish, or preparation of the manuscript.

We would appreciate the change in the online submission form.

3. Thank you for stating the following in the Acknowledgments Section of your manuscript:

> “The authors are also deeply grateful to all the women who participated in this work, and we hope that our efforts will improve their quality of life in all respects. The authors thank to INPer (approval number 212250-3120771), as well as their colleagues, Estela Y. Godínez-Martínez, Virginia E. Santillán-Palomo, María del Pilar Meza-Rodríguez, Carmen Flores-Cisneros, Cecilia Mota-González, Rosa Georgina Rodríguez-Delgado, and Edgar Bonilla-Reyes, for their excellent technical assistance, interest in this work, and helpful discussions.”

> We note that you have provided funding information that is not currently declared in your Funding Statement. However, funding information should not appear in the Acknowledgments section or other areas of your manuscript. We will only publish funding information present in the Funding Statement section of the online submission form. Please remove any funding-related text from the manuscript and let us know how you

would like to update your Funding Statement. Currently, your Funding Statement reads as follows: This study was supported by the Instituto Nacional de Perinatología (Mexico City) under approval number 212250-3120771.

Please include your amended statements within your cover letter; we will change the online submission form on your behalf.

Answer. We removed any funding-related text from the manuscript. About the Funder Statement, we would like to update as follows:

> "This study was supported by the Instituto Nacional de Perinatología (Mexico City) under approval number 212250-3120771. The founders had no role in study design, data collection and analysis, decision to publish, or preparation of the manuscript."

4. Your ethics statement should only appear in the Methods section of your manuscript. If your ethics statement is written in any section besides the Methods, please delete it from any other section.

Answer. The ethics statement has been removed; it should appear only in the Methods section.

5. When completing the data availability statement of the submission form, you indicated that you will make your data available on acceptance. We strongly recommend all authors decide on a data sharing plan before acceptance, as the process can be lengthy and hold up publication timelines. Please note that, though access restrictions are acceptable now, your entire data will need to be made freely accessible if your manuscript is accepted for publication. This policy applies to all data except where public deposition would breach compliance with the protocol approved by your research ethics board. If you are unable to adhere to our open data policy, please kindly revise your statement to explain your reasoning and we will seek the editor's input on an exemption. Please be assured that, once you have provided your new statement, the assessment of your exemption will not hold up the peer review process.

Answer. We appreciate the recommendation. Due to this, we are considering making the dataset available on a repository called Zenodo (https://zenodo.org/), once we submit the revised version back to PLOS One. A dataset uploaded to Zenodo is usually available almost immediately, and if a 'hold' period is set, the metadata will be made public immediately, but the files cannot be downloaded until the date you specify.

6. Thank you for providing your underlying data as Supporting Information.

We note that the data set contains text or data that is not in English. Please note that PLOS is an English-language publisher, so we require data sets to be provided in English as well. Please upload an English-language version of your data set.

This will also allow us to determine if your data follows PLOS standards per our Data Availability policy here: https://journals.plos.org/plosone/s/data-availability

Answer: Thank you for your feedback. We apologize for our carelessness and can confirm that the entire manuscript and all supporting information are now in English.

If the reviewer comments include a recommendation to cite specific previously published works, please review and evaluate these publications to determine whether they are relevant and should be cited. There is no requirement to cite these works unless the editor has indicated otherwise.

Answer. Your suggestion has been considered in the revised version of the manuscript.

---- Reviewer 1 ----

- **TITLE.**

  Short, concise, but misleading. The sentence “Quality of life associated with clinical and sociodemographic” judges the relationship. Please correct per the recommendation provided.

  Suggestions: “Predictors of the QoL among the postpartum period of women diagnosed with HIV”

Answer. We would like to express our sincere appreciation for the reviewer’s suggestion regarding the title. In our study, the objective was to examine the associations and impact of clinical and sociodemographic variables on quality of life, rather than establish predictive models. However, we recognise the rationale behind using the term 'predictors' –which included predictive analyses and their validation as predictors–, and referring to these variables as 'predictors' could misrepresent the scope and methodology. Therefore, to address both approaches, we included predictive analyses and revised the title to encompass both objectives of our study.

Regarding the term ‘women diagnosed with HIV’, since the mid-1990s, the term 'people living with HIV' has been used to distinguish chronic infection from AIDS (the advanced stage), reduce stigma and humanise patients, following the expansion of effective antiretroviral therapy. The terms 'infected' and 'sick' are considered stigmatising and technically inaccurate, as individuals receiving treatment may never develop AIDS. For this reason, we consider 'women living with HIV' to be a more precise and inclusive description.

In light of these considerations, we have revised the title to:

"Quality of life in the postpartum period of Mexican women living with HIV: The role of clinical and sociodemographic factors"

We believe this formulation better reflects the aims of our study, maintains academic neutrality, and provides a more accurate representation of the study population.

- **ABSTRACT** Follow the Plos One preferred headings of the Abstract.
- Should be revised per the corrected introduction, methods, and results.
- Write the conclusion instead of the discussion.

Answer. We have reorganised the abstract into the following sections: Introduction, Methods, Results, and Conclusions. The statements have been adapted accordingly to fit this structure. We also addressed the suggestion to replace the discussion with a clear conclusion. We hope you agree that this restructuring enhances clarity and aligns with the journal's requirements.

- **INTRODUCTION**
  - **The rationale is not clear. What is the progress of QoL? Due to what intervention? Has this intervention been considered in this study?**

Answer. In response to your initial comment that 'the reasoning is unclear', we have carefully revised the manuscript, rewriting several paragraphs in this section to improve clarity and coherence. We hope that these changes make the rationale of our study clearer. Please review from lines: 56-99.

Concerning the question 'What is the progress of quality of life? We interpret this as reflecting the impression that our study is longitudinal, involving repeated application of the QoL instrument (e.g. at baseline and post-intervention). However, we would like to clarify that this is not the case; our research is a cross-sectional study in which the QoL instrument is applied only once. No intervention is planned to compare pre- and post-intervention outcomes. Our objective is to quantitatively assess participants' perceptions of their well-being, and then to explore the potential impact of sociodemographic and clinical factors on these perceptions. In this way, our study aims to provide evidence that supplements the traditional clinical perspective by incorporating less visible yet equally important aspects of well-being.

- **METHODS AND MATERIALS**

  The methods section lacks essential reporting of the sampling techniques and the ART intervention, as follows:

  - The study design is not appropriately reported. With insufficient explanation of the ART intervention.

Answer. Regarding the description of the study design and explanation of ART, we would like to clarify that our research was an observational, cross-sectional study. The study did not involve any intervention beyond the standard of care by the institution. As all participants received ART as part of routine care, it was

considered a determining factor to analyse for its impact on quality of life, rather than an intervention.

To avoid ambiguity, we have strengthened the methodological description to explicitly state that the study is cross-sectional, with a single application of the QoL instrument. No intervention or longitudinal follow-up was performed. ART forms part of the standard treatment received by all participants and is analysed as a clinical variable possibly associated with QoL rating.

The text was amended at line 118 (Study design)

We hope that these clarifications address the concerns raised and provide a clearer understanding of the study design and scope.

- How was sampling conducted? Is it purposive, convenient, or what?

R= Sampling was conducted using a non-probabilistic convenience approach. Postpartum WLWH receiving ART treatment as part of the infection care, over 18 years old, with prenatal care at the INPer, were invited to participate during the visits of baby´s follow-up. The text was amended at lines 137-139. Women enrolment section.

- The inclusion criteria are not clear? Why was ART treatment included? Have you applied any measures to monitor and control the ART intervention?

Answer. Concerning the inclusion criteria, it is clarified that eligible participants were postpartum women living with HIV, aged over 18 years, who were receiving ART as a part of their infection care and had received prenatal care at the National Institute of Perinatology (INPer) and just came back only for their baby's follow-up until obtaining discharge, upon confirmation of no HIV vertical transmission (18 months). The selection of these criteria was driven by two overarching objectives. Firstly, it was deemed essential to ensure a homogeneous study population, thereby facilitating the analysis and enhancing the interpretability of the results. Secondly, the criteria were chosen to reflect the clinical reality of women routinely managed at INPer, ensuring the relevance and applicability of the findings to real-world scenarios. You can find in the section that corresponds to it in the Materials and Methods.

About the issue of the rationale behind the incorporation of ART, it is crucial to underscore that this was not an intervention initiated by the study; rather, it was the standard treatment that all participants were already receiving as part of their routine medical care. As a result, ART was considered a significant clinical condition and relevant contextual factor in the analysis, given its recognised influence on health outcomes in women living with HIV.

Finally, regarding the question of whether any measures were implemented to supervise or control ART, it is important to note that no additional monitoring or

intervention was performed within the framework of this study. The present study concentrated exclusively on the evaluation of quality of life and its possible influence from sociodemographic and clinical variables, without implementing any modifications or interventions in the participants' treatment.

- Please justify why the sampling was performed during pregnancy, and data collection was performed after delivery.

Answer. We appreciate your careful observation. The potential confusion may have arisen because woman living with HIV are identified during pregnancy at our institute or are transferred from other health institutions for prenatal care following an HIV diagnosis. However, enrolment was carried out exclusively in the postpartum period, when these women attended their infants' routine follow-up visits at INPer. Our research focused specifically on postpartum women, and therefore the invitation to participate was extended at that stage.

We have made the necessary corrections to the manuscript. These ensure that the enrolment process is described accurately and consistently with the study's focus on postpartum women. See Materials and Methods section.

- How was the ART intervention tracked?

Answer. We would like to clarify that ART was not an intervention assigned, introduced, or supervised by the study or the research group, but rather the standard treatment routinely provided to all women living with HIV at the National Institute of Perinatology (INPer) or the ART that patients referred from other medical institutions were already receiving. Accordingly, no specific measures were implemented within the framework of this research to monitor or control the administration of ART.

ART was therefore considered a clinical factor relevant to the analysis, but it was not modified, supervised, or controlled by the investigators, and this study is purely observational.

- Have you maintained follow-up of the women from recruitment to the actual data collection? if yes, how was the follow-up maintained?

Answer. We would like to clarify that no follow-up was conducted from recruitment to data collection. The study was designed as a cross-sectional assessment. Therefore, the quality of life instrument was applied only once,

during the postpartum period. This took place when women attended their infants' routine follow-up visits at the National Institute of Perinatology (INPer).

At that time, participants provided written informed consent, and their sociodemographic and clinical information was obtained from questionary and directly from their medical records. The study did not involve any longitudinal monitoring or repeated measurements, nor did it include supervision or control of antiretroviral therapy (ART) beyond the standard clinical care provided by the institution.

The objective of the study was to evaluate quality of life at a single point in time and to explore its possible association with sociodemographic and clinical factors. The study did not include the implementation or maintenance of follow-up procedures.

- What are the clinical determinants that have been evaluated for association with QoL?

Answer. Concerning the clinical determinants assessed in relation to quality of life. In our study, the following clinical variables were systematically considered: other chronic diseases, years since HIV diagnosis, presence of HIV-related symptoms, last CD4 count during pregnancy (cells/mm^3), initial viral load (copies/mL), history of HIV-infected children, current partner's HIV status, type of ART, adherence to ART as reported by the physician, intrapartum prophylactic ART, use of efavirenz during pregnancy, perception of perinatal complications, obstetric history, time since delivery (months), type of birth delivery, age at sexual debut, and number of sexual partners.

These determinants were selected to provide a comprehensive framework for understanding the multidimensional nature of quality of life in postpartum women living with HIV. It is important to note that our study population is distinct from many previous investigations, which often include only men, non-pregnant women, or mixed cohorts where men predominate. In such studies, the clinical variables most frequently analysed are limited to years since HIV diagnosis, presence of HIV-related symptoms, CD4 count, viral load, WHO clinical staging, sexual behaviour, and type of ART. All determinants or variables you can see in **S3. Table.**

In contrast, our focus on postpartum women necessitated the incorporation of additional reproductive and perinatal factors (e.g., obstetric history, perinatal complications, intrapartum prophylaxis, and postpartum timing), which are highly pertinent to this specific population. We believe that this broader set of determinants strengthens the analysis and allows for a more accurate assessment of quality of life in women living with HIV during the postpartum period.

- Wrong selection of the statistical test. With multiple independent variables and one dependent variable, it is advised to use multiple linear regression analysis rather than Spearman's correlation. In this case, as the QoL is categorized in nominal variables, you should apply the Multiple Regression Model.

Answer. In agreement with your recommendation, it was necessary to request authorisation from other departments at our Institute in order to consult and obtain the missing data. This ensured the completeness and accuracy of the information used. Secondly, we considered the recommendation to carry out a multiple regression analysis; however, within the current study design, this would only be feasible if the dataset did not contain tautological relationships (i.e., the dependent variable is a consequence of the independent variables, expressed as VD = V11 + V12 + V13 + … Vn). In this case, the issue arises because the central research question is which of the independent variables best predicts quality of life – in essence, which component best predicts the sum of its parts. The consequence of this is that the model produces overfitting, yielding a correlation value of one. This is not a valid result.

To identify variables most closely related to Quality ofLife (QoL) and evaluate their performance as potential predictors, a multinomial logistic regression (MLR) analysis was conducted on the complete dataset. This analysis adhered to all model assumptions, using "high quality of life" as the reference category. Variables exhibiting an odds ratio (OR) > 2 were selected for further consideration. Additionally, a Principal Component Analysis (PCA) was performed on the entire dataset to identify variables accounting for 60% of the total variance. The results of the MLR and PCA were cross-referenced; variables with an OR > 2 and an absolute loading value ≥ 0.6 were then tested for multicollinearity, with a Variance Inflation Factor (VIF) < 5 and a Tolerance < 0.7 required for inclusion. All statistical analyses were performed using SPSS Statistics 26 (IBM, NY, USA). Please note that the above information can now be found in the manuscript, in the Materials and Methods section, in the statistical analysis subsection, line 189.

The results derived from this new analysis have been incorporated into the corresponding sections of the manuscript, Results, line 359, and Discussion.

We believe that this methodological modification strengthens the study's rigor and lends greater solidity to the conclusions, as it allows for a more precise identification of the influence of sociodemographic and clinical factors on the quality of life of the population analysed.

- **RESULTS**

- Page 11: lines 203-204: “32% of our population were diagnosed with HIV during their current pregnancy, and most of the cases they believed that it was sexually transmitted Please justify the prolonged data collection period, about 18 months, considering the small sample size. This might induce balance or lack validity of the data.” Please justify that the study targets women with HIV receiving ART treatment, and the 32% of the sample were diagnosed with HIV?

Answer. As a response to your inquiry, some technical factors and aspects of the research objectives contributed to the extended data collection period. According to our institution's statistics, between 30 and 35 pregnant WLWH arrive at our institution for perinatal care each year. Therefore, if all participants were willing to take part in the study, we could have reached the required sample size within a year and a few months. Secondly, the data collection period began after the initial lifting of the nationwide lockdown in Mexico due to the COVID-19 pandemic, as our institution strictly adhered to safety protocols, given the heightened risk for pregnant women with the virus. Visits by women living with HIV during the postpartum period were inconsistent, prompting the clinic to arrange group appointments for their infants' check-ups. However, numerous appointments were not attended, leading our institution's ethics committee to recommend an LTFU=50% in the sample size. Thirdly, to assess the quality of life (QoL) of patients living with HIV during the postpartum period and also investigate the possible association of clinical and sociodemographic variables influencing this, the increased sample size was utilized to achieve these objectives.

To respond to the question. The study included women living with HIV (n = 75), 32% of whom received their diagnosis during the current pregnancy (n = 24). The remaining patients had already received their diagnosis before admission to our institution, having been transferred from other healthcare institutions where they had received treatment for HIV. Regardless of whether they were referred or had been recently diagnosed, all patients received antiretroviral treatment in accordance with national guidelines for the management and treatment of people living with HIV (PLWH). Most of the patients were admitted during the third trimester of pregnancy. Those who had been diagnosed more recently were managed in accordance with the guidelines for pregnant women living with HIV. If they had previously been assigned an ART treatment by the issuing institution, this was maintained without changes due to the proximity to childbirth. As this is a cross-sectional study rather than a longitudinal study, efforts were made to ensure that the data collection schedule covered the entire follow-up period for the infants (18 months of life) at the facility, to confirm that vertical transmission of HIV had been avoided.

- S2 file, Table. Justify collecting the QoL scores at different postpartum times of postpartum-WLWH.

Answer. The postpartum times during which the QoL instruments were administered correspond primarily to the period of follow-up for their babies at our institution and to the visits during which their babies undergo ELISA testing to confirm that there was no vertical transmission of HIV. This period lasts up to 18 months. At the time of the final test, the baby is discharged from follow-up care, and the mothers are given a discharge letter confirming that there was no vertical transmission of HIV. We believe that follow-up during this period is emotionally important for them, as WLWH are often plagued by worry and guilt regarding their babies' health.

---- Reviewer 2 ----

- **The minimum sample size calculated is 42, how the recruitment of participant is 75 was not explained**.

Answer. We acknowledge that the initial sample size calculation (adjusted for 50% loss to follow-up) indicated a sample size of n=42 participants. However, during data collection, we observed significant variation in the QoL scores obtained among WLWH. This finding highlighted the importance of conducting a more robust analysis to identify the sociodemographic and clinical factors associated with QoL. We therefore decided to expand recruitment to 75 participants in order to strengthen the statistical power of these analyses. In fact, a post-hoc calculation of the sample size for a multiple linear regression analysis, considering 15 variables that showed statistical significance in a Spearman's correlation analysis, yielded a required sample size of n=74.8 participants to achieve intermediate but acceptable power, using Cohen's f^2 as the effect size measure. Thus, the inclusion of 75 women ensured that our study had sufficient power to explore the complex interaction of factors influencing the QoL of this vulnerable population, whilst also improving the reliability and applicability of our findings. You can see this in line 128 in Study design.

- **The results/table shows n=75, with many variables showing varying sample size, indicating varying missing data that may have been cleaned before the analysis**.

Answer. Following your observation and recommendation, it was necessary to request authorization from other departments at our Institute in order to consult and obtain the missing data. This ensured the completeness and accuracy of the information used. We present the updated results in this revised version of the manuscript. You can see this update on Table 1, Table 2, Figure 2, and in the Result and Discussion sections.

- **Table 4 (QoL in the women) may be presented as a figure (Pie or Bar Chart) instead as a table.**

Answer. Thank you for the feedback. We have presented Table 4 and Table 5 in a figure format to improve the presentation of results.

---- Reviewer 3 ----

1. **The introduction is written in a complicated manner; the remainder of the manuscript is more clearly written and easier to understand (compared to the introduction).**

Answer. We appreciate your feedback. We have reviewed this section, considering the comments you kindly provided below, and have incorporated all of them.

3. **Line 41:**

a) Where the authors propose that the QoL questionnaire be included in the healthcare system, i.e. where in the postpartum care algorithm or even the antepartum system that QoL questionnaire be included? Is there a plan to share the findings with health authorities?

Answer. Findings of our study justify incorporating the QOL questionnaire into both prenatal and postpartum services at our Institute. It is important to note that the instrument itself is not intended to replace existing clinical protocols, but rather to complement them by providing a structured assessment of patient-reported outcomes.

We propose integrating the QoL questionnaire at two critical points of care:

i) Antepartum care: After the first or second routine prenatal consultations at the HIV Care Clinic for Pregnant Women.

ii) Postpartum care: At standard follow-up visits with their babies after delivery.

The HIV Care Clinic for Pregnant Women at our institute has the capacity to implement activities that strengthen patient care, and QoL monitoring is a feasible and informative way to monitor this population.

Finally, although the sample size of 75 participants may seem small, it accurately reflects the epidemiological situation at our Institution. Only 30–35 pregnant women living with HIV transferred or diagnosed at our National Institution each year. This scarcity is due to the dual vulnerability of being both pregnant and living with HIV, which makes this population particularly complex and valuable for study. Unlike regions with a higher prevalence, such as certain areas of sub-Saharan Africa, the number of cases in Mexico City is considerably lower. Consequently, while the generation of findings may be limited, this study provides valuable insights into a highly vulnerable and underrepresented population. Furthermore,

ongoing research at our institute focuses on measuring quality of life and identifying the sociodemographic and clinical factors adversely affecting it among women living with HIV during pregnancy and the postpartum period. The sample size was designed to achieve statistical significance. In this regard, we adapted the conclusion for better understanding in line 48.

4. **Lines 59-60: The link between stigma and discrimination and chronicity of infection is unclear. Perhaps the authors could rather frame the stigma and discrimination as an additional challenge.**

Answer. We have carefully revised the manuscript, rewriting several paragraphs in the Introduction to improve clarity and coherence. You can read in lines 56-70, as follows:

From a clinical perspective, PLWH are confronted with the chronic nature of the infection, the long-term effects of ART and the associated challenges of adherence. They also experience the burden of non-infectious comorbidities (7). In addition to these clinical demands, PLWH also face persistent stigma and discrimination. This should be understood as an additional challenge that compounds the complexity of living with HIV, rather than being a direct clinical manifestation (2). Together, these clinical and social factors have a significant impact on perception of Health and QoL (8,9). This impact is particularly pronounced in highly vulnerable groups, such as WLWH, who may encounter additional stressors during pregnancy, the postpartum period, and motherhood (10). During these periods, pain and physical discomfort, combined with environmental factors, social relationships and personal beliefs, can exacerbate vulnerability and perpetuate stigma and discrimination. Consequently, perceptions of health status deteriorate, QoL declines and negative outcomes such as reduced care and diminished attention may ensue (12).

5. **Lines 61 a 67: This sentence is very difficult to follow, and the authors are advised to replace the one sentence with 2-3 linked sentences.**

Answer. This paragraph was rewritten and replaced in several sentences. You can read in lines 63-70.

This impact is particularly pronounced in highly vulnerable groups, such as WLWH, who may encounter additional stressors during pregnancy, the postpartum period, and motherhood (10). During these periods, pain and physical discomfort, combined with environmental factors, social relationships, and personal beliefs, can exacerbate vulnerability and perpetuate stigma and discrimination. Consequently, perceptions of health status deteriorate, QoL declines, and negative outcomes such as reduced care and diminished attention may ensue (12).

6. **Lines 75 a 79: This sentence should precede the previous 2 sentences for ease of reading.**

Answer. This paragraph was rewritten and was reorganized as your suggestion for better understanding, see lines 71-86.

There are also significant associations between maternal postpartum health (both mental and physical) and the health of their children [13]. Therefore, a mother's QoL could influence her children's QoL as they grow up. It is known that even in non-complicated perinatal period important physical and emotional changes occurs, and factors such as age, parity, social and economic issues, obstetric complications, obesity, history of alcohol dependence, sleep difficulties, stress, anxiety and postnatal depression affect the mother QoL [13–15]. Furthermore, studies assessing QoL in mothers with chronic diseases have reported that their children also experience lowest QoL [13,16]. Another study evaluating health-related quality of life (HR-QoL) in mother-child pairs from low-income settings found that these children had poorer outcomes and behavioural disorders compared to children in the general population [17]. It is important to emphasise that the traditional approach to health has primarily focused on the clinical dimension, centered on the detection, treatment and cure of diseases. However, health cannot be reduced to the mere absence of illness as it permeates every aspect of a patient's life, making this approach insufficient. Therefore, the assessment of QoL emerges as a valuable indicator of self-perceived well-being and health status, encompassing broader dimensions of QoL [18].

7. **Line 80: "Affects" may not be the correct word for what the reviewer understands the authors wish to convey- would "encompass" not be a better word?**

Answer. When the sentence is paraphrased, the problem is solved.

8. **Line 82. Review the document for TENSE- this should be "has."**

Answer. The change has been applied, you can see in line 87.

9. **Lines 90-94. Please consider simplifying the sentence extending from.**

Answer. The sentence was simplified as your request and was moved to line 100 for better understanding.

Globally, WLWH represent 53% of all PLWH [22], and the majority are of reproductive age. This increases the likelihood of pregnancy and the risk of exposing their newborns to HIV vertical transmission [23]. Around 1.2 million WLWH become pregnant each year, and 84% of these (72-98%) receive ART to prevent mother-to-child-transmission (MTCT) [23].

10. **Line 95. Incorrect spelling ("focussing").**

Answer. The sentence is paraphrased and the word was removed. You can see in line 94.

Expert groups on HIV/AIDS have proposed to include the QoL evaluation in PLWH as a measure of therapeutic success, as well as a 'fourth 95' HIV target which should be achieved [8]. QoL is increasingly recognised as a key component of public health [21],

11. **Lines 103- 107: difficult to follow, please simplify.**

Answer. The paragraph has been simplified. You can see in line 107-113.

Significant progress has been made in understanding the QoL of pregnant and postpartum WLWH in Latin America (LATAM), particularly in Brazil [24–26]. Nevertheless, important gaps remain that could be addressed by future research, particularly by moving beyond a purely medical perspective. There is a need for greater exploration of factors such as emotional impact, social stigma and support networks, as well as the population characteristics that shape these experiences. This is essential to better understand how these women navigate their condition and the demands of motherhood.

12. **Line 116: what is meant by "this stage"?**

    Answer. The word "this stage" was eliminated, you can see in line 124.

13. **Line 119: this is an unusually high LTFU- is this based on previous LTFU rates when working with this particular group of women?**

Answer: This study evaluated the quality of life (QoL) in this population (n = 28). The loss adjustment in this study was calculated at 50%, as suggested by the research and ethics committees of this institution. This is because, once the pandemic began in 2020 and extended until 2022, following up with this population became difficult. Other studies conducted in our institution on this same population have shown lower loss percentages (less than 10%), which may be sufficient for achieving the study's objectives.

14. **Line 120: Please advise why 75 women were recruited if the final sample size was calculated as 42.**

Answer: We recognize that the initial sample size was calculated as 42 patients, with an allowance for a 50% loss rate. However, during data collection, we identified significant variations in the scores obtained from the WLWH. This finding emphasized the importance of conducting a robust analysis to identify the sociodemographic and clinical factors associated with QoL. For this reason, we decided to increase recruitment to 75 participants in order to strengthen the statistical power of these analyses. Subsequent multiple linear regression

analysis revealed that a total of 15 variables were significant in a correlation analysis (Spearman's rho). The required sample size for intermediate but acceptable power was 74.8 participants, using Cohen's f^2 as the effect measure. Therefore, including 75 participants ensures that the study has the appropriate power. The complex interaction of multiple factors influencing the QoL of this vulnerable population may improve the reliability and applicability of our findings.

15. **Line 126: please elaborate on how the participants' anonymity and privacy were protected**

Answer. The anonymity and privacy of the participants were strictly protected throughout all phases of the study. Immediately after the QoL assessment tool was applied and sociodemographic and clinical data were obtained from the participants' medical records, they were each identified using a unique alphanumeric code. This coding system replaced any personal data, such as names or medical record numbers, meaning that the dataset used for analysis contained no information that could directly identify the women. The coded files were stored on secure systems with restricted access solely for the research team, and all analyses were performed on anonymized databases only. Thus, the confidentiality and privacy of the participants were strictly guaranteed from data collection through to statistical analysis.

16. **Line 149: Please confirm the reference for the classification of participant perception of QoL as poor, medium and high.**

Answer. The classification used in our study does not come directly from the WHOQOL-HIV BREF instrument manual, as this does not provide cut-off points for categorizing QoL as high, intermediate or low. Instead, our proposed stratification is based on the approach adopted by several African researchers who have used the mean total score of the instrument to distinguish between high and low quality of life (e.g. Girma et al., 2023; Abebe et al., 2018). We consider it appropriate to follow this approach because it enables each population to act as its own benchmark, accounting for the cultural and contextual factors that influence perceptions of quality of life. We acknowledge that other authors have used fixed intervals of the original score (4–20 points), including Alexander and Thomas (2022), Oliveira-Alexander et al. (2017), Da Costa and Oliveira (2013) and Morandi dos Santos et al. (2007). However, these studies do not explicitly explain the methodological justification for these cutoffs, creating some heterogeneity in the literature. In our case, the decision to adopt the African reference stems from the need to interpret the results in a way that is more aligned with the reality of the population under study, while acknowledging the cross-cultural validity of the instrument and accepting that categorization may vary depending on the context.

Bibliography:

- Alexander, T., & Thomas, A. (2022). *World Journal of AIDS*, 12(2), 87–98. https://doi.org/10.4236/wja.2022.122008
- Oliveira-Alexander, et al. (2017). *Enfermería Global*, 16(4), 571–582. http://dx.doi.org/10.6018/eglobal.16.4.267571
- Da Costa, C., & Oliveira, D. (2013). *Revista de Enfermagem da UFPE*, 7(10), 5925–5933. https://doi.org/10.5205/reuol.4377-36619-1-ED.0710201305 (doi.org in Bing)
- Morandi dos Santos, et al. (2007). *Revista de Saúde Pública*, 41(suppl 2), 131–137. http://www.scielo.br/pdf/rsp/v41s2/en_5947.pdf
- Girma, S., et al. (2023). *Heliyon*, 9(2), e13318. https://doi.org/10.1016/j.heliyon.2023.e13318 (doi.org in Bing)
- Abebe, A., et al. (2018). *BioMed Research International*, 2018, 5965343. https://doi.org/10.1155/2018/5965343

We included the bibliography in line 166.

17. **Line 150: Please advise on the reasons for the modification of the instrument and if modifications were made to all 11 questions. Further, please also advise on the total number of questions following the modifications.**

Answer. The WHOQOL-HIV-Bref questionnaire was applied in its entirety and without modification. The first eleven introductory questions and the remaining 31 questions that make up the instrument itself were not altered in terms of wording, structure or content. The only modification was the addition of a supplementary block of 11 sociodemographic questions, separate from the original instrument. These questions are not part of the WHOQOL-HIV-Bref, but they were included to provide a more detailed interpretation of the results by obtaining information directly from the participants, since the data recorded in electronic files may have changed since their admission to the Institute and the commencement of their medical care and treatment. It was considered methodologically appropriate to collect this information at the same time as applying the instrument to ensure the consistency, homogeneity, and currency of the sociodemographic data, thereby avoiding potential gaps or discrepancies in the clinical records. The additional sociodemographic questions did not affect the structure or validity of the original instrument. You can read lines 167-172.

18. **Lines 152- 153: The sentence is incomplete.**

Answer. We have modified the sentence following the observation to improve its clarity and flow. You can read lines 169-170.

19. **Line 178: Please consider discussing the reasons for late antenatal presentation discussed, especially if linked to QoL.**

Answer. Thank you for the recommendation. We include in discussion section. In Mexico, delayed prenatal care remains a critical public health concern, disproportionately affecting vulnerable populations. Disparities are most pronounced among women with lower educational attainment, those residing in rural areas, and individuals of lower socioeconomic status—inequities that were further exacerbated by the COVID-19 pandemic. You can read lines 405-409.

20. **Line 193: slightly misleading statement- might be clearer if the sentence read as "reported smoking (4%) etc" as in its current phrasing it reads as 17% denied knowledge and then admitted to smoking/ alcohol etc in their partners.**

Answer. Yes, this paragraph can be confusing, so it has been rewritten for clarity, replacing the previous version, lines 234-239, and now reads as follows: Concerning the partner's drug addiction, 17% of participants denied knowledge of their current partner's substance use. The remaining 83% of the sample reported smoking (3%), alcoholism (12%), illegal drug addiction (7%), combined alcohol and drug use (14%), and a mix of alcohol, smoking, and drugs (5%) among those aware of it. Regarding tattoos and piercings, 25% had tattoos, 5% had piercings, and 9% reported having both (Table 1).

21. **Line 204: the word "they" is unnecessary.**

Answer. That word was crossed out in line 246 of the manuscript.

22. **Line 209: Is this only for the newly diagnosed women or for all participants?**

Answer. We were referring to all the participants. This information was included in the manuscript, line 252, as follow:

During birth, 76% of all participants received ART intrapartum as prophylaxis, based on clinical criteria and in accordance to the national guidelines for the care of WLWH [32].

23. **Line 212: How were the 3 categories of adherence determined?**

Answer. The adherence categories were determined by the specialist physicians who treated the participants and documented in the medical records. The majority of participants exhibited "high" adherence to treatment. This was based on undetectable viral loads and adequate CD4+ cell counts in this cohort. You can see line 254.

24. **Line 212: Were these complications in the perinatal period related to the ART regimens?**

Answer. The perinatal complications reported were unrelated to any use of antiretroviral treatment. This information was described in the line 256 in the manuscript.

25. **Lines 236- 238: Is this an interpretation of the results in table 3?**

Answer. Yes, that was an interpretation of Table 3, so it has been removed from this line of the manuscript.

26. **Line 252: please review the sentence construction for the sentence beginning "There were not..."**

Answer. This paragraph has been rewritten and replaces the previous one in the manuscript, please review the updated results in lines 305-312.

27. **Line 253: please clarify which group of postpartum women is being referred to**

Answer. Following the rewriting of the paragraph referred to in item 26, This paragraph has been rewritten and replaces the previous one in the manuscript, please review the updated results in lines 305-312.

28. **Line 307: The word "officially" implies that it is endorsed but not yet included in the targets. This 4th target, however, is still under debate and perhaps the authors should consider rephrasing this to indicate that some experts have advised a fourth target etc.**

Answer. We concur with your assessment of the fourth target. This paragraph has been revised to incorporate your feedback. The following changes have been made to the manuscript and you can see in line 395-398.
Notwithstanding, the compelling counsel proffered by numerous experts in the field, the 2030 95-95-95 targets established by UNAIDS notably exclude the 'fourth 95' target, which stipulates that 95% of people living with HIV (PLWH) should attain a QoL that is comparable to that of individuals who are HIV-negative.

29. **Line 316: With 92% living in urban areas, please confirm that there is a lack of transport in these areas.**

Answer. We can confirm that, despite the presence of fully urban residential areas in the suburbs of Mexico City, the lack of adequate and affordable public transportation that is both safe and affordable is a daily reality for these women. Therefore, the paragraph was revised to incorporate your observation in the

manuscript, lines 410-419, and now reads as follows: These women reside in marginalised suburbs of Mexico City, where there is a deficiency in access to adequate, high-quality public transportation at an affordable price. Furthermore, the safety of these areas is compromised by the high crime rates that prevail. Women’s reliance on transport systems is fundamental to their economic participation and social integration. However, inadequate accessibility, spatial constraints, and pervasive safety concerns significantly restrict their mobility, producing forms of social exclusion. The absence of secure and reliable travel options not only shapes women’s perceptions of the urban public realm but also curtails their access to education, employment, and healthcare. Consequently, mobility and safety emerge as critical determinants of gender equity and broader societal inclusion.

30. **Line 316: Please advise how the lack of transport and security "results in discrimination, gender and social inequality and economic dependence."**

Answer. We addressed this question in the previous issue (29).

31. **Line 319: Please advise what is referred to by the word "which thrived." Is it adherence that is being referred to? How was this recorded in their clinical files?**

Answer. We are referring to the fact that the high adherence that the doctors reported for the participants—and which was documented in their medical records like “high adherence”—was maintained or persisted until the time of delivery. For this reason, we replaced the word "thrived" with "persisted" in that same section of the manuscript, line 426.

32. **Line 326: The authors should consider rephrasing this as 47% were aware of their partners serostatus and 26% were unaware of their status. In this 26%, some partners may be seropositive so it is advised that the authors be careful in how they present this information**.

Answer. We concur with your point. Consequently, the paragraph has been revised and now reads as follows in the manuscript, line 434:

In our sample, 75% of the participants are aware of their partners' HIV status. It should be noted that some of these individuals are HIV-positive.

In addition, the table 2 presenting these results has been modified.

33. **Line 332: Perhaps the authors would like to consider combining these sentences.**

Answer. We prefer to rephrase both statements, keeping the word count nearly the same, so that the manuscript reads as follows, line 441:

The QoL instrument exhibited excellent internal consistency, aligning with evidence from the English version validated in Ethiopia and the Spanish version.

34. **Line 343: The part of the sentence starting with "considering that India..." does not link well with the first part of the sentence. Please review.**

Answer. We have revised the paragraph to incorporate your comment, and we believe it now reads more clearly. In the manuscript, the previous text has been replaced with the following, lines 452-455:
A study in India, found higher QoL scores, among non-pregnant-WLWH, despite their poverty. The study attributes this result to a structured program for the distribution of ART, close monitoring of adherence, and strong community support. It is important to note that India has a lower HDI (0.644) compared with Mexico.

35. **Line 351: Please review the sentence starting with "And"- perhaps this is not meant to be a capital letter?**

Answer. This was corrected in the manuscript by removing the period and adding a lowercase “a” to the word “and” in line 464.

36. **Lines 373- 385: The reviewer acknowledges the evidence presented in relation to the SARS-CoV-2 pandemic. Perhaps the authors could explore the QoL scores post 2020 and if there was improvement, to compare with other studies that might have assessed this.**

Answer. We will include a statement to the effect that, while WLWH faced greater challenges during the pandemic (as evidenced by the literature), the problems inherent to their HIV infection persist and are the primary factors affecting their well-being. Measuring QoL in a post-pandemic era is a subject of interest to us, and work is currently underway in this area. Please find in lines 524-527 the manuscript's content: For WLWH, the post-lockdown era signifies more than a mere return to normality during pregnancy and postpartum. The situation is still significantly impacted by a combination of ongoing psychosocial, structural, and HIV-related factors, and efforts have been made to address their emotional and psychological wellbeing and persistent stigma.

37. **Line 400- 403: The reviewer advises the authors to be cautious in this statement- the sample size is small and specific to one institution.**

Answer. The statement to which the reviewer refers in this line has been rewritten and replaces the previous one in the manuscript, line 592, reading as follows: The sample is representative of the Mexican postpartum WLWH who were treated at the antenatal care at INPer. The participants are from Mexico City and its suburbs, (the Estado de México).

38. Line 405: Consider reworking this sentence to read better.

Answer. Please be advised that the sentence in question was changed when the entire paragraph was rewritten (lines 601–604). As stated in the manuscript, the following is written: Furthermore, the call is made for the inclusion of QoL evaluation in WHO strategies, the fourth 95 target, and government and societal efforts to reduce stigma, foster acceptance, and improve environments for WLWH, future generations, and exposed newborns.

39. Feedback on Tables included in the manuscript:

a. In Table 1:

I. Under monthly income- please confirm that the first line is meant to read "Depends on his parents." The table presents characteristics of the women on study.

Answer. Yes, we refer to "Mexican postpartum WLWH as financially dependent on his parents". To clarify this term, we change it to – none[a] –, and in the footer of Table 1 you can find this definition.

II. Please advise on the relevance of sexual debut, number of sexual partners, sexual abuse history and tattoos in the context of this study.

Answer. These factors are significantly relevant in the context of WLWH and their QoL because they often intersect as indicators of higher risk behavior, vulnerability, and stigma, impacting both mental and physical health. These factors are key determinants of HIV acquisition and play a major role in their psychosocial well-being. You can find these subjects in the discussion section, lines 446-449.

b. Table 2:

I. Was VL analysed in relation to duration of ART? 68% of participants have been diagnosed for more than 3 years.

Answer. We completed Table 2 with the medical records information about ART therapy during pregnancy and postpartum. We can see that ART therapy decreases the VL and many of them become undetectable close to the delivery.

II. Please clarify why 17 women did not receive intrapartum ART- were they already on treatment? Why were 77 women eligible for intrapartum ART with 68% diagnosed with HIV infection 3 or more years prior?

Answer. To address this question, it is essential to consider the complexity of medical care for WLWH patients during pregnancy and childbirth. A significant

number of patients do not seek medical care at our institution until the third trimester of pregnancy. Some patients are referred from other institutions with an established HIV diagnosis and already on antiretroviral therapy, while others are admitted for prenatal care and are diagnosed at our institution. Specifically, for these 17 women, all were on antiretroviral therapy, and according to their medical records, most had been living with the infection for more than 3 years, demonstrated high adherence to their antiretroviral therapy, and in all cases had an undetectable viral load. Therefore, they were eligible to receive intrapartum antiretroviral therapy (ART). In the case of those who did receive intrapartum ART, this group was more heterogeneous. Some had recently been diagnosed, others showed moderate or low adherence, some had a detectable viral load, and others had a combination of two or more of these factors. According to institutional protocols, intrapartum ART was necessary in these cases.

III. What does the perception of perinatal conditions refer to?

Answer. We refer to the perception of WLWH about perinatal complications as high-stakes events, bleeding, prolonged labor, threatened miscarriage, premature rupture of membranes, gestational diabetes mellitus, growth restriction, and the health of the newborn due to prematurity, neonatal respiratory distress, and others. We include this information in Table Footer 2.

c. **The heading of table 4 is incorrect as all women are included in the table, not only the women with poor QOL.**

Answer. We replaced Figure 1 instead of Table 4, and the mistake in the heading has been fixed.

We thank to the Editor and reviewers in advance for your understanding and support, and we look forward to your feedback soon.

Best regards

MSc. Jessica Hernández Pineda
Department of Infectology and Immunology
National Institute of Perinatology
Phone:+015555209900, ext.497
jesspinq@yahoo.com.mx
